# The Rationale of Pulp Extirpation

**Published:** 1905-04

**Authors:** Hart J. Goslee

**Affiliations:** Chicago


### THE RATIONALE OF PULP EXTIRPATION.[1]

BY HART J. GOSLEE, D.D.S., CHICAGO.

In assuming to interest the members of this distinguished body, I must confess that, even with all the latitude so kindly granted, the selection of a subject which would be even partially commensurate with the honor conferred has occasioned me no little concern.

A discussion of pulp extirpation has suggested itself to me for this occasion, for at least two specific reasons. First, because of the radical change in the method of procedure whch has been adopted in the past few years, and secondly, because the topic is just now a particularly live one, made so by the more or less discordant notes of warning and of enthusiastic commendation, which are the echoes of the hour.

Lest any one should imagine that it might be my intention to carry him to the realms of a purely scientific discourse in the presentation of this subject, let me at once dispel such a thought, and affirm that the more selfish motive of personal benefit prompts the effort, rather than the expectation of adding new light or particularly original thought to the subject.

And yet, conceding that true progress is to be attained mainly by scientific research and discourse, may we not at the same time contribute to its development by now and then interspersing what some of us are pleased to term "practical" views? I believe we may.

The present popularity of pulp extirpation is doubtless very largely due to the somewhat diversified opinions held by many of our most prominent men.

Any radical departure from the time-trodden paths of successful operative procedure must be productive of just such a condition, for the reason that it will at once appeal particularly to two classes of those who go to make up the whole, each class of which, for the good of the profession be it said, is ever alert and active,—the one eagerly grasping at all things new, and quickly bounding away on the wings of enthusiasm, and the other seeking to restrain such a flight with the bridle of conservatism.

---

[1] Read before the Academy of Stomatology, Philadelphia, February 28, 1905.

Thus the seeming idiosyncrasies of the human mind, portrayed by such widely diversified opinions and experiences as are observed in men engaged in the pursuit of similar lines of effort, such as ours, and particularly of those who are equally and pre-eminently successful, is an interesting study. And yet, in reality, when we analyze these "seeming idiosyncrasies" we can only deduce that they but emphasize the infallibility of personal equation, for whilst it is true that we all seek knowledge from the same fountain-heads, and that all who are honest and conscientious work to the same end, yet the means employed with success by one would meet only with dismal failure in the hands of another. This is personal equation, and must enter into the lives and work of all men, else we would soon become mere automata.

With the advent of cocaine and pressure—or so-called "pressure anæsthesia"—for the removal of pulps from teeth, the former procedure seemed to bid fair to be almost completely revolutionized, and hence we now find the operation a source of much variety of opinion and discussion. On the one hand we hear an enthusiastic cry for the abandonment of a method which has been continuously and successfully employed for half a century and more, and a plea for the universal adoption of the newer and more radical method; on the other hand, we hear in words of caution of the ill-results and failures recorded against this newer procedure.

A perusal of the pages of our recent literature can but confirm this statement, and must excite in those who have not followed the subject closely almost every feeling from surprise to consternation, if there be any intervening. If this is true,—and I think it quite beyond denial,—should we wonder at disquietude and confusion existing in the minds of that vast majority of the profession, and particularly of the younger men, who look to, and in a large measure depend upon, those who essay to teach and write for their enlightenment, encouragement, and progress?

Or need we marvel at, or ask the occasion for, disquietude or confusion existing in the minds of many concerning this particular subject when we read from a lecture recently delivered by our distinguished friend, Dr. B. Holly Smith, of Baltimore (*Items of Interest*, January, 1905), wherein he says, *literally*, that arsenic for the purpose of devitalizing the pulps of teeth should be relegated to the practices of mediæval dentistry; that its action is positively not limited to the confines of the tooth itself; that when it is applied to the pulp it will go on and result in a destructive process of the tissues contiguous with the end of the root; and in which he then recapitulates by making the broad and sweeping statement that he can prove that every tooth in which arsenic has been sealed for the purpose of devitalizing the pulp will be a source of trouble sooner or later.

If these be facts, is it not a strange coincidence that the profession should only now awaken to a realization of such alarming or dangerous possibilities, after having universally used arsenic since the days of Drs. Greenwood and Spooner in 1837, or thereabouts?

Furthermore, in contradistinction to these and other similar views, we read a paper by Dr. R. Ottolengui, of New York (*Dental Cosmos*, September, 1904), in which is recorded a series of observations and experiences in connection with this newer method,—cocaine and pressure,—the results of which tend to indicate so large a proportion of failures and bad after-effects produced by its employment as to, at least, offer anything but encouragement to those who might otherwise have been influenced by the remarks of the gentlemen previously quoted, to abandon the use of arsenic.

Verily, therefore, the proposition from this view-point needs must appear to be somewhat perplexing, and yet since it becomes necessary to remove pulps, we must, at the present time, at least, either go back to the now ancient and barbarous practice of driving them out with wooden points, resort to the slower process of strangulation and destruction by the use of arsenic, or else adopt the more immediate process of pressure anæsthesia.

While nothing perhaps short of mental aberration would now induce any one to consider the former crude and heroic practice, still it did not serve its purpose, and, in the light of past experience, and of calm, rational judgment is there not virtue in *both* of the latter methods? Personally I indulge in the firm conviction that there *is*, and that each method has its respective sphere of usefulness, and will be found manifestly advantageous in proportion as it may be judiciously selected, and its technique mastered and carefully observed.

As progressive practitioners of a modern science it is our patent duty to study and analyze all methods by which the comfort and convenience of the patient, and in turn of the operator, may be increased or facilitated, and to select and adopt those which in our judgment seem to offer the most favorable opportunities for obtaining the desired results in the case at hand, for, after all, while we may speculate at random, theorize at will, and become "faddists" if we are so inclined, it is nevertheless *results*, pure and simple, that we are after.

That the application of arsenic to the pulps of teeth has caused trouble, and may to-day cause trouble, is indisputable. Still, do not the records of the past, and does not the experience of the present, indicate that such instances are the exceptional rather than the common result?

Notwithstanding the statements previously quoted, nor questioning the sincerity of their author, it is my belief, based entirely upon observation and experience, that more pericemental trouble has resulted from the use of cocaine in the past four or five years than was ever produced by arsenic, and that "lame" teeth are more prevalent to-day, as a result, than they were prior to the introduction of cocaine and pressure for the purpose of removing the pulp.

The troublesome manifestations produced by the use of arsenic may be classified as complex and simplex in character, and the distinction thus drawn would include as complex those wherein pericemental trouble developed and lameness ensued, while those designated as simplex would embrace only that class in which the disturbance is confined to the pulp itself, and hence only of a temporary nature.

In analyzing the cause and effect in the class designated as complex it is but rational to conclude that such physiological disturbances may be attributed to any one of three general sources: first, to the use of too large a quantity of arsenic; second, to allowing it to remain sealed within the tooth for too long a period; or third, to the abnormal enlargement of the foramen. The last condition, however, would include so small a proportion of cases as to make them exceptional, and thus not warrant further consideration, for pericemental trouble would be very likely to ensue from the application of either arsenic or cocaine, because of the difficulty of confining the action of either drug to the pulp itself.

But as regards the former, if one-fiftieth of a grain of arsenic or less be sufficient to destroy the vitality of the pulp, then it is, of course, obvious that the use of a greater quantity simply means that the further absorption of any excess, above the actual requirements, must but increase the action, and in turn the destructive process, and thereby invite trouble beyond the apex.

And further, if twenty-four, or forty-eight, or even seventy-two hours is sufficient time to admit of the absorption of this quantity of arsenic, and of producing a physiological change of the pulp-tissue, then a longer period of time would also only promote a more unlimited action of the drug and the probability of apical disturbance.

Therefore, since the pulp of a tooth is capable of absorbing within itself only a given quantity of arsenic, in proportion as the minimum quantity of the drug is sealed within the tooth and allowed to remain but the minimum period of time will the best results obtain and the percentage of subsequent pericemental troubles decrease; and yet, logical as this may seem, how few practitioners observe it as carefully as might be, or make any distinction, in regard to the quantity used, between a tooth having a large pulp and one with a small one, or between any of the teeth in the denture, or discriminate as to the vital resistance of the various physiological types.

Those troublesome manifestations, which have been classed as being simplex in character, while frequently attributed to the mere presence of arsenic, are doubtless often due to an unwise application, or to faulty technique, and may usually be directly traced to placing the drug in a tooth in which the pulp is already highly inflamed or congested, or to undue pressure produced in sealing the cavity.

Since it is a well-known fact that this drug only acts as a powerful irritant to tissues undergoing any stage of the inflammatory process, it is obvious that its application in these conditions must be preceded by such palliative treatment as will at least temporarily relieve the inflammation and reduce the congestion. Thus, in proportion as the condition of the pulp approaches the normal, will the application of arsenic increase in effectiveness and the susceptibility to its irritating influence be correspondingly decreased.

Furthermore, whenever the physiologically irritating influence of this drug is supplemented by any degree of mechanical irritation, such as may be, and as is so often, produced by undue pressure in sealing the treatment within the tooth, it is manifestly easy to account for the resultant trouble.

In this particular connection Dr. D. D. Smith, of Philadelphia, I believe, has called attention to the fact that arsenic should never be sealed in close proximity with the pulp, and that if the cavity be of such extensive proportions as will prevent an observation of this precaution, it should first be temporarily filled and the application of the arsenic then made by drilling accommodation for it in some other place in the crown more removed from such close proximity, which procedure, I think, is to be highly recommended in many instances.

While it must be conceded that many of the, perhaps, superficially apparent objections to arsenic may be eliminated by a more careful observation of the technique incident to its use, I believe that the primary cause of more subsequent pericemental troubles—and consequently of lame teeth—may be directly attributed to imperfectly filled canals as a result of the employment of such agents as tannic acid for the purpose of shrivelling or shrinking the pulp-tissue prior to its removal, than may be attributed to the possible continuation of the process of destruction resulting from the use of arsenic.

I am well aware of the fact that this practice is recommended, and has been for a long time more or less general, and yet, I believe that its employment only adds to the difficulties encountered in completely removing pulps so treated, and in subsequently thoroughly filling the apices of the canals. In fact, the theory that the "tanning" or "toughening" of the pulp-tissue by the use of a solution of tannic acid and glycerin, or other like agents used for the same purpose, *facilitates its removal* has, like the various theories of pulp mummification, proved to be only a delusion and a fallacy in my hands, and I believe that if pulps *may ever* be removed *en masse* with any degree of facility the procedure may be accomplished with more reasonable certainty immediately after the pulp has been desensitized than at any other time.

And again, and aside from this, there is another very pronounced objection to the use of the glycerite of tannin, to which the attention of the profession has been called. This pertains to the possible discoloration of the tooth which may result, and which in many cases can doubtless be directly attributed to the well-established fact that tannic acid and iron are chemically incompatible, and that when the former is placed in contact with the iron contained in the hæmoglobin, tannate of iron—a black ink—is the resultant compound.

While my former statement might possibly be construed as being an argument in favor of the cocaine and pressure method, and while it is so to a degree, still, irrespective of the possibilities of discoloration subsequently mentioned, the points which I desire to especially emphasize are these: that the pulp should be removed as soon as possible after it has been completely desensitized; that, owing to its fibrous nature, it may be removed *en masse* with greater certainty at this time than at any other; that if it is thus completely removed the opportunities for maintaining asepsis and thoroughly filling the entire canals are materially increased, and that if the canals are thus filled the possibilities of subsequent trouble will be likewise diminished, irrespective of whether arsenic, cocaine, or a combination of both methods be used.

Indeed, a combination of both, followed by *immediate removal,* is preferable to this so-called *shrinking* method, and may often be used to good advantage, for the reason that the hypersensitiveness which sometimes remains after the removal of the arsenic, is usually not sufficiently acute to preclude the further enlargement of the cavity until adequate access for the employment of cocaine and pressure may be obtained; and for the further reason that a more or less thorough mechanical sterilization of the cavity may thus be effected.

And now let us consider this question of sterilization. Undoubtedly a large proportion of the cases recorded by Dr. Ottolengui, and noted by myself and others, in which pericemental trouble followed the use of cocaine by the pressure method, may be attributed to imperfect sterilization of the cavity previous to its application.

This would lead us to agree with Dr. J. P. Buckley, of Chicago, in concluding that the question of cavity sterilization as applied to the use of cocaine in this manner is an important factor, and that whether it be obtained by mechanical or chemical means, it must always obtain.

In the use of arsenic, we usually find after its removal that the dentine is so desensitized as to admit of complete mechanical sterilization by means of the bur, but since such a procedure is rarely possible immediately preceding the application of cocaine by pressure, and since the dentine is usually permeated by micro-organisms and their poisonous biproduct, we must first obtain thorough chemical sterilization, or otherwise force these ptomaines through the tubuli into the pulp, and possibly into the apical tissues, when pressure is applied.

As some of these ptomaines are volatile liquids of an extremely irritating character, if the pressure employed be sufficiently great to force them through the apices and into the apical space, an acute inflammation of these tissues may be established as a direct result of this virulently irritating influence. Or as cocaine is itself a protoplasmic poison, which is admitted by pharmacologists, it may be that many of these disturbances are the direct result of the action and physiological incompatibility of this drug.

Thus as we study how to effectually sterilize the dentine prior to the application of this method; how we may best succeed in forcing our anæsthetic solution, and what it may possibly carry ahead of it only into the pulp-tissue and not into the apical space, and as we learn how to judiciously select our cases, may we employ cocaine and pressure, I think, with a minimum of bad results.

*Technique.*—Therefore, in my opinion, the successful application of this method demands, first, thorough sterilization of the cavity; secondly, the employment of a sterile solution of cocaine; thirdly, favorable opportunity for the proper application of pressure; fourthly, an observation of the precautions incident to the removal of the pulp and the filling of the canals, and fifthly, a degree of good judgment in the selection of cases.

*Sterilization.*—Excepting in that class of cases where the natural crown of a tooth which is to be used as an abutment for bridge-work is free from caries, and where the extirpation of the pulp may be demanded by the mechanical requirements in connection with the accurate adjustment of an artificial crown, to which this method is most applicable and usually preferable; or, in other words, wherever caries is present, and natural asepsis does not obtain, an absolutely thorough sterilization of the cavity is demanded.

Whilst this may be materially aided by first mechanically excavating the cavity to the fullest possible extent, still, owing to the sensitiveness of the dentine, it may usually be effectually accomplished only by chemical means.

Of the many agents used and recommended for this purpose, the desired results may be obtained to the best advantage, perhaps, by first isolating the tooth with the rubber dam and then flooding the cavity with a 1 in 200 solution of sublamin, or a 1 in 500 solution of bichloride of mercury, preferably the former, after which the excess should be first absorbed with cotton or bibulous paper, and the cavity then thoroughly dehydrated with alcohol.

*Cocaine.*—As commercially prepared solutions of cocaine are not stable, in order that the second requirement—that of always employing a sterile solution—may obtain to the best advantage, the crystals should be finely pulverized and dissolved with sterilized water in each individual case. While carbolic acid, adrenalin chloride, and other agents are recommended for this purpose, a sterilized water is preferable because of the fact that in the event of being forced through the apex of the tooth—as it is in many cases irrespective of the care used to prevent—less physiological disturbance of the contiguous tissues will be produced.

Ordinary distilled water would, of course, answer the purpose to a degree, but a sterilized or essential oil water is preferable because of thus being absolutely and permanently sterile, and of the probable diminution of troublesome manifestations obtained by its sterility.

*Pressure.*—As already intimated, no portion of the technique of this procedure is regarded as being of much greater significance than that relating to the proper application of pressure, and as extreme care must be exercised in employing only sufficient pressure to force the solution through the dentine and into the pulp-tissue, I am of the opinion that the various mechanical devices designed for this purpose—in the use of which absolute control of the pressure may not be easily maintained—are therefore dangerous, and that the best results will obtain from the more simple and less heroic methods.

These simpler methods may be employed effectually if means for confining the solution of cocaine within the tooth are observed, and where the shape, location, or proportions of the cavity are not favorable, they may usually be made so either by the employment of matrices or by the use of cement. Indeed, cement will be found indispensable in many classes of cavities, and may be used either to dam up the walls of the cavity, or to previously entirely fill it, subsequently drilling through the filling until the dentine is reached and exposed, through which opening the cocaine and plunger may then be placed and the pressure applied.

It is also my belief that in all instances the pressure should be gentle at first and cautiously increased until a slight response on the part of the patient, which is usually manifested, is noted, when the plunger should be held firmly without increasing or diminishing the pressure for a few moments. After the pain has subsided the pressure may then be gradually increased until no response is observed, which will usually indicate that the cocaine has reached the pulp and that anæsthesia has been effected.

*Removal of Pulp.*—As soon as anæsthesia is thus indicated an exposure of the pulp should be attempted. If this is not possible, an effort should be made to approach as closely to it as may be done without inflicting pain, and then again resort to further effort with cocaine, until a full and complete exposure may be effected. Ample access should then be secured by enlarging the cavity to the extent of the dimensions of the pulp-chamber, and the pulp immediately removed by mechanical means.

*Controlling Hemorrhage.*—And now we come to a very important phase of the subject,—the treatment of the more or less copious hemorrhage which usually follows, and the proper time for the filling of the canals.

While adrenalin chloride, hydrogen peroxide, and many other hæmostatic or oxidizing agents are recommended for the control of the hemorrhage and the evacuation of the blood, it is my belief that no effort to control it should be made, that it should be allowed to bleed freely, and that the blood should be evacuated from the canals only by such mechanical means as tepid water or shreds of cotton or bibulous paper twisted on the broach, or by some agent, such as alcohol or chloroform, which will dissolve the blood, for the reason that such a procedure relieves the congestion in the apical area, and is an important factor in preserving the color of the tooth. In this connection every precaution should be taken to remove the blood from the canal and cavity, and not to chemically decompose it within the tooth, as is done when hydrogen peroxide, for instance, is used for this purpose.

Indeed, it is claimed by Dr. Buckley and others that to the use of agents which will oxidize the iron in the hæmoglobin of the blood remaining within the tooth may be attributed to a large extent the subsequent discoloration; and, furthermore, that any discoloration may be avoided by an observation of these precautions.

*Filling Canals.*—As soon as the hemorrhage may be checked in the manner indicated it is manifestly safe to fill the canals, but unless absolute certainty of their being perfectly dry exists, it is doubtless the best and most conservative practice to seal some healing, antiseptic agent, such as creosote, within them, and defer the filling until a subsequent sitting. This also allows for the natural healing of the disrupted vessels and thereby diminishes the cause and possibilities of the development of pericemental troubles.

*Indications and Contraindications.*—While, in line with my original argument, the indications for the employment of arsenic are more or less general, yet the same may scarcely be said of this newer method.

Indeed, it must be conceded that the application of cocaine and pressure is particularly contraindicated in at least three general classes of cases: first, in those cases mentioned by Dr. Ottolengui, where the presence of calcific deposits within the tooth retards the penetration of cocaine, and thus precludes or acts as an impediment to the complete anæsthetizing of the pulp; second, that class of cases where the decay is so extensive as to leave the pulp without sufficient protection to withstand the necessary pressure, and yet where it is also not possible to puncture it, at least, perhaps without administering a general anæsthetic; and third, those cases where the remote, unfavorable, or inaccessible location of the cavities, the hypersensitiveness of the dentine, or the physical condition of the patient preclude its use.

In otherwise favorable conditions, however, it will be found most advantageous, and particularly in the extirpation of congested pulps which are giving trouble, and where an exposure may first be obtained, and in teeth the crowns of which are entirely free from or but slightly attacked by caries, and in which the pulps may consequently be more or less normal.

In conclusion, let me summarize by reiterating that both methods possess virtue, and are useful in their place when employed with a proper observation of the prerequisites essential to success, and that, to a very large extent, the selection of the one most applicable to the individual case will be a question the solution of which good judgment alone will dictate.

